# Towards Measuring Stress with Smartphones and Wearable Devices During Workday and Sleep

**DOI:** 10.1007/s12668-013-0089-2

**Published:** 2013-05-08

**Authors:** Amir Muaremi, Bert Arnrich, Gerhard Tröster

**Affiliations:** Wearable Computing Lab, ETH Zurich, Gloriastrasse 35, 8092 Zurich, Switzerland

**Keywords:** Stress, Smartphone, Sleep, Wearable device, Heart rate variability

## Abstract

Work should be a source of health, pride, and happiness, in the sense of enhancing motivation and strengthening personal development. Healthy and motivated employees perform better and remain loyal to the company for a longer time. But, when the person constantly experiences high workload over a longer period of time and is not able to recover, then work may lead to prolonged negative effects and might cause serious illnesses like chronic stress disease. In this work, we present a solution for assessing the stress experience of people, using features derived from smartphones and wearable chest belts. In particular, we use information from audio, physical activity, and communication data collected during workday and heart rate variability data collected at night during sleep to build multinomial logistic regression models. We evaluate our system in a real work environment and in daily-routine scenarios of 35 employees over a period of 4 months and apply the leave-one-day-out cross-validation method for each user individually to estimate the prediction accuracy. Using only smartphone features, we get an accuracy of 55 %, and using only heart rate variability features, we get an accuracy of 59 %. The combination of all features leads to a rate of 61 % for a three-stress level (low, moderate, and high perceived stress) classification problem.

## Introduction

Work-related stress is the response that people have when presented with work demands and pressure which are not matched to their knowledge and which challenge their ability to cope [5].

In the modern, fast-paced society, work overload is more and more common. In 2007, stress was identified to be the second most common work-related health problem in the EU [13]. We all are tempted to try to do more in less time, without giving much thought to the consequences. However, exposure to continuous high workload over a long period of time without sufficient recovering can often lead to physical exhaustion and prolonged negative affect and even more serious conditions such as chronic stress disease [7]. At the same time, excessive workload represents a major reason for employees quitting their jobs, which results in high economic costs for the companies [36]. It is therefore of great interest to monitor the affect changes of employees.

The most common method to quantify positive and negative affect is simply asking people about their mood in an interview or by letting them fill in questionnaires. There are standardized methods to measure such affect changes and, specifically, those that help quantify stress. Examples of such questionnaires are the Perceived Stress Questionnaire [11] or the Depression Anxiety and Stress Scale [21].

In contrast to the common method, in this paper, we investigate the potential of a modern smartphone and a wearable heart rate monitor for assessing affect changes in daily life. We derive features from the smartphone sensor data during workday and heart rate variability (HRV) measurements from a chest belt worn during sleep. As ground truth, we gather self-assessments on perceived positive and negative affect during working days. We use smartphone features and HRV measures as predictors for building classification models to discriminate among low, moderate, and high perceived stress.

In the smartphone market, the two most prominent mobile operating systems are iOS and Android. The vast majority of the users participating in our trial have iPhones, and since the idea is to let the users use their own smartphones in a normal way, we decided to build our system for smartphones based on iOS. The Wahoo (http://www.wahoofitness.com) chest belt, capable of measuring the HRV, was chosen because of its iOS support.

The rest of the paper is organized as follows: In the next section, related work is presented. Next, the used questionnaire for self-assessment is shown, followed by the explanation of the extracted features and the modeling of the stress score. The experimental setup is described, and the cross-validation results are discussed in the evaluation section. The paper is concluded by summarizing the main achieved results and by giving some improvements and extensions planned to be done in the future.

## Related Work

Stress recognition remains one of the main research topics in the area of affective computing [2]. However, the focus has shifted from controlled experiments to real-life scenarios out of the lab. Along this direction, mobile devices such as smartphones and mobile biosensors, mainly skin conductance sensors (see, e.g., [15]), have become the main tools for analysis.

### Stress Recognition using Smartphones

Searching in the app stores for “*stress*,” one can find more than 1,000 related apps in the Apple market and much more in the Google market. These apps can be categorized into:

- *Diaries*: Collect and aggregate subjective ratings
- *Guides*: Tips and tricks on how to deal with stress; some are combined with diaries
- *Relaxations*: Support of relaxation exercises, like breathing techniques to calm down
- *Sensor measures*: Sensor-based tracking of behavior related to stress

In conclusion, nowadays, the majority of related apps follow the common approach of asking and providing textual description on how to deal with negative affect or stress.

However, there is emerging research on tracking of behavior related to negative affect and stress, based on sensors. For example, the recently introduced smartphone application “BeWell” [18] monitors three daily types of behavior: physical activity, sleep pattern, and social interaction. In addition, the app provides visualizations of the measured behavioral aspects. For example, the amount of physical activity is visualized by the swimming behavior of an animated fish: in case of low physical activity, the fish swims very slowly, and in case of high physical activity, the fish performs fast loops. The system was evaluated with a small set of five users over a short period of 1 week. The three behavioral aspects are treated separately, and there is no approach given to derive one single well-being score. “AMMON” [8] is a speech analysis library for analyzing affect, stress, and mental health directly running on the mobile phone. This library is limited to speech and is tested using an emotion corpus [37]. The recognition accuracy is 93.6 % for the two-class problem: stress increase vs. stress decrease. Similar to that, “StressSense” [22] recognizes stress from human voice using smartphones in real-life conversational situations. The reported accuracies are 82.9 % for the indoor scenario and 77.9 % for the outdoor scenario. “MoodSense” [20] tries to infer the users’ mood using SMS, e-mail, phone call, application usage, web browsing, and location data. Here, the audio part is missing and the subjects are a group of students. The user mood can be inferred into four major types with an average accuracy of 91 %. A similar work is presented in [4], where GPS, WiFi, Bluetooth, phone calls, and SMS logs are used to detect specifically stress-related changes in user’s behavior. Here, again, audio is not considered and the number of seven students is very limited. The system is able to detect an average behavior modification of 53 % for each participant during the exam time. From all solutions, MoodSense is the only one developed for iOS systems. It uses the “LiveLab” [35] library able to collect sensor data in the background (similar to the Android sensing framework “Funf” [3]). The crucial problem here is that this library requires the iPhone to be “jailbraked” (the Apple policy is broken), a fact that makes the solution to be not acceptable for most people.

In our work, we focus on all sensor modalities available on a regular iPhone. The 35 subjects under investigation are employees of three IT companies, and the evaluation is done for 4 months within the subject’s real working life.

### Stress Recognition using HRV

HRV reflects the variation of the beat-to-beat (RR) intervals. HRV is known to be an indicator of the autonomic nervous system activity [38]. In many studies, HRV measures were employed to investigate mental disorders or responses to stress. For example, in [23], the phases of bipolar patients are characterized by means of HRV features obtained with a sensorized T-shirt. In this clinical state assessment, features are reported that show significant differences across bipolar states. In [17], HRV patterns were found that allow to identify subjects which report a high stress experience. The classification between the high and the low stress groups is 66.1 % accurate. In most examples, a stimulus that invokes stress is presented to the subjects. In [10], e.g., people work under a controlled cognitive load and under time pressure. In [39], the HRV features are evaluated during emotional visual elicitation, and in [26], the HRV features of students under stress due to university examination are investigated. The accuracy of discriminating students under stress from situations without stress is 90 %. Beside the HRV analysis during the day, there is also research done in investigating HRV patterns recorded during sleep as a supplement of the day analysis. However, these studies concentrate on a specific illness, such as bipolar disorder, e.g., [28], or obstructive sleep apnea, e.g., [41], and do not treat the problem of stress in general.

In our work, we investigate HRV patterns from normal healthy people during sleep. In order to do that, we use an unobtrusive HRV measuring device that can be easily worn at night.

## Approach and Methods

**Fig. 1:**
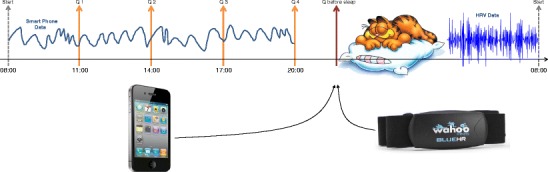
One full data collection cycle and the questionnaires shown during the day

In general, we follow the approach of estimating changes of subjective self-perception of stress using smartphone sensor measures and information derived for the HRV signal during night. From 8 a.m. to 8 p.m., the day is divided into four sections, and randomly within each section, a notification is shown which asks the user to fill in a self-assessment questionnaire. In parallel to that, smartphone data are being collected during the day in the background. Before going to sleep, the user answers an additional stress question and puts on the Wahoo chest belt which collects HRV data during night until the next morning. After getting up, a new cycle of data collection begins. Figure 1 shows schematically one such full data collection cycle. The idea now is to use these smartphone and wearable device data to estimate the self-assessment stress score.

In the following, we describe the subjective assessments, the features extracted from the objective data, and the models used to predict the stress level.

### Questionnaire and Audio Response

The user of the app is asked to fill in a Positive and Negative Affect Schedule (PANAS) questionnaire [40] four times per day between 8 a.m. and 8 p.m. This self-assessment questionnaire originally consists of 20 items. In the deployment phase of the app, we received complaints about the difficulty of answering to all of the items. To avoid any misunderstandings and to make the answering easier for the user, we reduced the questionnaire to the following 10 items: relaxed, tired, happy, stressed, concentrated, sleepy, interested, active, angry, and depressed (five PA items and five NA items). The questions are answered by moving a scrolling bar to the left for a low value and to the right for a high response value.

**Fig. 2:**
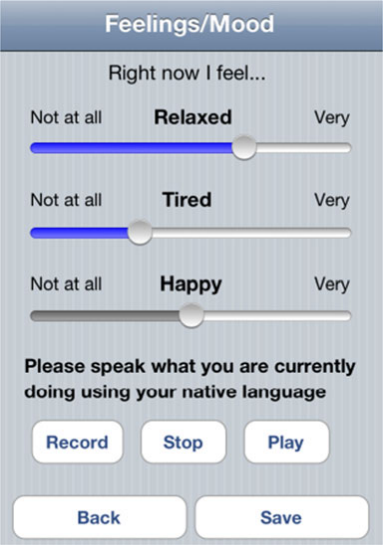
The implemented PANAS questionnaire reduced to 10 items: relaxed, tired, happy, stressed, concentrated, sleepy, interested, active, angry, and depressed (five PA items and five NA items). The questions are answered by moving a scrolling bar resulting in a continuous response value. The last question asks the participants to respond verbally about what he is currently doing in his native language. The voice is recorded by pressing the Record and the Stop buttons

Beside answering to the PANAS questionnaire items, the user is asked to provide a voice message in which he speaks about what he is currently doing using his native language. The voice recording is performed by pressing a start and an end button. In this way, the privacy aspect is not a critical point since the user is conscious that his voice is being recorded. The questionnaire is shown in Fig. 2.

The last action the user actively performs with the phone is answering to the stress self-assessment question before going to sleep: “How stressful have you felt today?” The person is supposed to think in a retro-perspective way about the passed day and to rate it by moving a scrolling bar from very relaxed to very stressed resulting in a continuous stress score between 0 and 1. This type of asking questions about the feelings in hindsight corresponds to the daily reconstruction method [16], as opposed to the experience sampling method [19], i.e., current perception of stress.

In this study, we concentrate only on the single stress score of the night.

### Signal Processing Chain

**Fig. 3:**
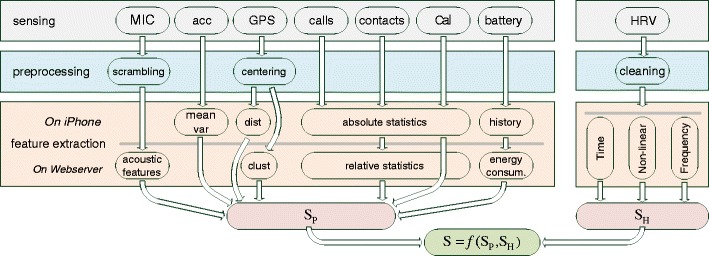
Signal processing chain: from raw sensor data to the final stress score

An overview of the signal processing chain of the app is depicted in Fig. 3. In order to reduce the battery consumption, the accelerometer and the GPS sensor are sensed only every 5 min for 30 s. The microphone signal is accessed as part of the self-assessment questions. The contacts (address book) and the calender events are read once before the data are uploaded to the web server. The current battery level is stored in 5 % intervals, and a call event is registered as soon as the phone call happens. The computed RR intervals on the Wahoo device are sent in real time to the smartphone which then continuously stores the values locally during the whole night.

### Features

We follow the approach of collecting as much smartphone data as possible, extracting features based on state-of-the-art research and trying, to find the best feature set with respect to cross-validation accuracy at the end. Tables 1 and 2 show the complete extracted feature list.

**Table 1:** Smartphone feature list

Category	Sensor	No. of features
Audio	Microphone	>384
Physical activity	Accelerometer	2
	GPS	2
Social interaction	Phone calls	5
	Address book	3
	Calendar	4
	Battery	1

**Table 2:** HRV feature list

Category	Features	No. of features
Time	Sleep duration	1
	Mean RR, SDNN	2
	RMSSD, pNN50	2
	HRV index, TINN	2
Nonlinear	ApEn	1
	SD1, SD2, SD1/SD2	3
Frequency	LF, HF, LF/HF	3

#### Smartphone Features

The smartphone features are divided into audio, physical activity, and social interaction features.

##### Audio

To anonymize the recorded speech, the audio file is sliced into small chunks and these slices are randomly permuted within each second, resulting in a very low speech intelligibility. The speech of the person becomes not understandable, while at the same time, the performance of the acoustic analysis of the speech is not degraded. The open source library “openSMILE” [14] is used for the calculation of the audio features. The selection of features is driven by the feature set proposed in the INTERSPEECH 2009 Emotion Challenge [34].

##### Physical Activity

Dependent on the environment, type of activity, and health of the person, the physical activity may influence the perceived stress level, both in a positive and in a negative way (see, e.g., [29, 32, 33]). We estimate the physical activity using independently the device acceleration and GPS traces. The accelerometer features are the mean value and the variance of the magnitude of the device acceleration. The total distance traveled during the day and the number of locations visited are calculated using GPS. Locations are derived using the density-based clustering algorithm DBSCAN [12]. To anonymize the GPS traces, the absolute positions are shifted such that the centroid of the locations becomes the zero point of the coordinate system.

##### Social Interaction

An important factor of stress is personality traits. It has been shown that neurotic people have difficulty in managing stress [25]. A list of social interaction data derived from smartphones that are used to analyze the personality traits is given in [9]. From call events, we use number of calls, sum of all call duration, mean value and variance of call duration, and the ratio between incoming and outgoing calls. Number of events, total time spent in events, mean value of event duration, and the mean size of notes are extracted from calendar data. The absolute numbers of the address book are not relevant, but the relative changes of the number of contacts, phone numbers, and e-mail addresses could be more interesting. And, as an indication of battery usage, the ratio between the time the battery is not charging and the time the battery is charging is calculated.

#### HRV Features

Before deriving any features from the HRV signal, the RR intervals that differ more than 20 % from their predecessors are discarded. These samples are considered as outliers which may result from movements of the upper body during sleep or any heart beat anomaly such as ectopic beats. The selection of the HRV features and its classification into three groups is motivated by the review article given in [1].

##### Time Domain Features

The time domain features include sleep duration, mean value of RR intervals (mean RR), standard deviation of RR intervals (SDNN), root-mean-square successive difference of RR intervals (RMSSD), number of successive difference of RR intervals which differ by more than 50 ms expressed as a percentage of total RR intervals (pNN50), and two geometric measures, namely the total number of RR intervals divided by the height of the histogram of all RR intervals measured on a scale with bins of 1/128 s (HRV index) and the triangular interpolation of RR interval histogram (TINN).

##### Nonlinear Features

Approximate entropy (ApEn) measures the complexity or irregularity of the signal. Large values of ApEn indicate high irregularity, and smaller values of ApEn indicate a more regular signal [39]. SD1 represents the fast RR variability in the HRV data, while SD2 describes the long-term variability (SD1 and SD2 are also known as the coefficients of the Poincoir plot). And, SD1/SD2 is the ratio of short interval variation to the long interval variation [1].

##### Frequency Domain Features

The power spectral density of the RR intervals is estimated using the Lomb–Scargle periodogram [31] since this algorithm can deal with time series which are not necessarily evenly spaced. The spectrum is divided into three frequency bands: very low frequency (VLV), 0.01–0.04 Hz; low frequency (LF), 0.04–0.15 Hz; and high frequency (HF), 0.15–0.4 Hz. The features used are the normalized values of LF, HF, and the ratio of LF and HF (LF/HF). The ratio LF/HF is not only useful as a feature for detecting stress but is also very important to differentiate between the sleep stages [27].

### Stress Score Modeling

S_P_ is the stress score using only the extracted smartphone features during the day, and S_H_ is the score using the HRV features of the night. The stress score S combines the two individual scores S_P_ and S_H_. For the estimation of the scores, we use multinomial logistic regression (logit) models.

The binomial logit is defined as

1 $$ \operatorname{logit}(p_{i}) = \beta_{0,i} + \beta_{1,i}\cdot x_{1} + \ldots + \beta_{m,i}\cdot x_{m} = y_{i}, $$

with $i=\{0,1\}$ and the logit function defined as

2 $$ \operatorname{logit}(p_{i}) = \ln\left(\frac{p_{i}}{1-p_{i}}\right). $$

$\beta _{0,i} \ldots \beta _{m,i}$ are the *m* regression coefficients for the class *i*, and $x_{1} \ldots x_{m}$ are the *m* variables or predictors of the linear regression. The probability of the class *i* is

3 $$ p_{i} = \operatorname{logit}^{-1}(y_{i}). $$

The binomial case is extended to the multinomial case with three classes, $i=\{0,1,2\}$. The multinomial logit model assigns the input variables $\textbf {x} = [ x_{1} \ldots x_{m}]$ the class *c* with highest probability:

4 $$ c(\textbf{x}) = \arg\max_{i\in\{1,2,3\}}{(p_{i})} \in\{0,1,2\}, $$

where $p_{0} + p_{1} + p_{2} = 1$. For more details, see, e.g., [24].

**Fig. 4:**
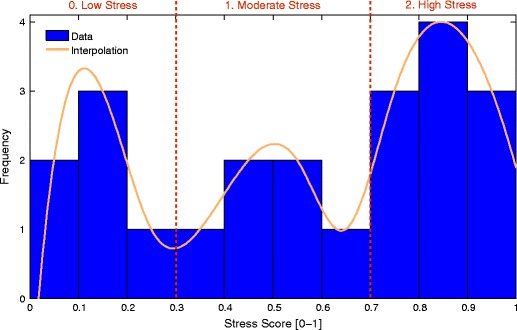
The stress score distribution of one exemplary user

In our case, the input variables $\textbf {x}$ are the features, and the model parameters $\beta $ are estimated using training data. The motivation for choosing a three-class model is shown in Fig. 4. The typical stress score distribution can be roughly segmented into three regions, which in our case correspond to three ordinal classes:

- 0 (low stress), if stress score $\leq $ 0.3,
- 1 (moderate stress), if $0.3 <$ stress score $< 0.7$, and
- 2 (high stress), if stress score $\geq $ 0.7.

#### Daily Stress Score

The daily stress score DS is a continuous value between 0 and 1 and reflects the stress level of the previous day. This score can also be seen as the acute stress level of a person. DS_P_ and DS_H_ are the individual scores using smartphone data and HRV data. They are computed as

5 $$ \text{DS}_{\text{K}} = [p_{0\text{K}}, p_{1\text{K}}, p_{2\text{K}}] \cdot [0, 0.5, 1]^{T}, $$

**Fig. 5:**
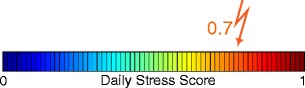
An example of the visualization of DS_P_ for the class probabilities $\{p_{0\text {P}}, p_{1\text {P}}, p_{2\text {P}}\}$ = $\{0.15, 0.3, 0.55\}$

$\text {with K} \in \{\mathrm {P},\mathrm {H}\}$. Figure 5 shows an example of the visualization of DS_P_ for the class probabilities $\{0.15, 0.3, 0.55\}$. If training data from both modalities are available at the same time, then a common logit model is trained with features from both smartphone and HRV, and DS is computed as

6 $$ \text{DS}_{\text{K}} = [p_{0}, p_{1}, p_{2}] \cdot [0, 0.5, 1]^{T}, $$

where $\{ p_{0}, p_{1}, p_{2} \}$ are the outcome probabilities of the common model with the input $\textbf {x} = [\textbf {x}_{\text {P}} ,\textbf {x}_{\text {H}}]$. However, in a practical case, a common trained model is not available, if for daily training, data from one modality are missing. In that case, DS is computed using DS_P_ and DS_H_ as

7 $$ \text{DS} = w_{\text{P}} \text{DS}_{\text{P}} + w_{\text{H}} \text{DS}_{\text{H}}. $$

$w_{\text {P}}$ and $w_{\text {H}}$ are the a priori weights which correspond to the normalized classification accuracies of DS_P_ and DS_H_.

#### Long-Term Stress Score

**Fig. 6:**
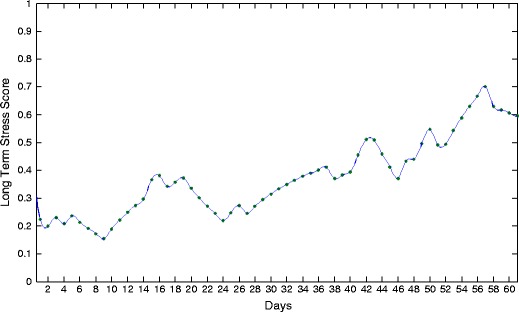
An exemplary profile of LTS over a period of 2 months

The long-term stress score LTS is a continuous value between 0 and 1 and estimates the chronic stress level of a person. Using a first-order low-pass filter, LTS at day *d* is updated according to the rule

8 $$ \text{LTS}_{d+1} = \text{LTS}_{d} + \alpha \cdot (c([\textbf{x}_{\text{P}}, \textbf{x}_{\text{H}}])/2- \text{LTS}_{d}), $$

with the filter coefficient $\alpha $ indicating the maximum change of LTS that may occur from day *d* to $d+1$. $c([\textbf {x}_{\text {P}}, \textbf {x}_{\text {H}}])$ is the output class of the common logit model using as input all features at day $d+1$. If either of the modalities is missing, then $c([\textbf {x}_{\text {P}}, \textbf {x}_{\text {H}}])$ is reduced to $c(\textbf {x}_{\text {P}})$ or $c(\textbf {x}_{\text {H}})$. In case the common trained model is not available for the classification, then $c([\textbf {x}_{\text {P}}, \textbf {x}_{\text {H}}])$ is modified to $c^*(\textbf {x}_{\text {P}}, \textbf {x}_{\text {H}})$ as

9 $$ c^{*}(\textbf{x}_{\text{P}}, \textbf{x}_{\text{H}}) = \arg\max_{i\in\{1,2,3\}}{(q_i)} \in\{0,1,2\}, $$

with $q_{i} = w_{\text {P}} p_{i\text {P}} + w_{\text {H}} p_{i\text {H}}$. The initial value LTS_0_ is the average of the daily stress scores DS during the training days. Figure 6 shows an exemplary profile of LTS over a period of 60 days with $\alpha = 0.1$.

## Evaluation

In this section, the experimental design of the conducted user study is first explained. Then, aggregated smartphone data over more than 1 day are shown for one specific user as an example. The best feature subset for each modality is determined, followed by the cross-validation analysis using these features. The section is concluded with a discussion part.

### Study Design

For our experiment, 35 users working in three IT companies participated for 4 months in the period between end of May 2012 and end of September 2012. The occupation of the subjects ranges from software developer to CEO of the company. The ages are equally distributed from 25 to 62 years. Eleven participants are female.

The participants had either iPhone 4 or iPhone 4S. The app was installed on their own devices in order to be able to use the smartphones in the usual way. The app can run on iPhone 4s at maximum for 12 h and on iPhone 4 for 14 h. The participants were equipped with Wahoo chest belts, which they used to collect HRV data during night. Table 3 summarizes the statistics of the collected data.

**Table 3:** Data statistics

Number of users	35
Number of days	127
Number of PANAS filled out	1,672
Number of HRV recordings	245
Number of Audio recordings	958
Amount of data (MB)	875

### Visualization of the Data

**Fig. 7:**
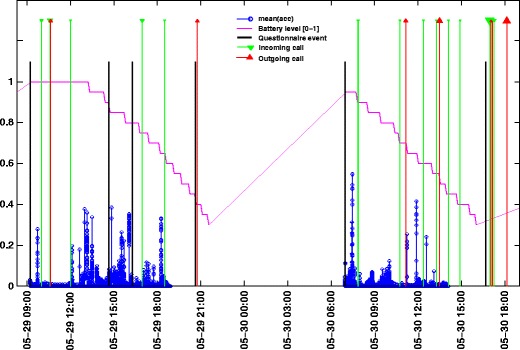
Data visualization of one user over 2-day time span. The size of the triangle indicates the call length

Phone calls, questionnaire events, motion, and battery level can be visualized on a plot such as shown in Fig. 7, where data from 2 days of one exemplary user are depicted. The battery level graph indicates that there is data gap between the 2 days. When the battery level falls below 50 %, the accelerometer is disabled, and when the battery undergoes the 30 % threshold, the GPS sensor is disabled which forces the app to be inactive and to disable the data collection in the background. Data collection is reactivated when the battery exceeds that threshold again and the app is put in the foreground, by either directly opening the app or when the user clicks the next notification message which asks him to fill in the next PANAS questionnaire. Beside that, the background sensing is stopped when the user starts an HRV night session and is reactivated when the user wakes up in the morning.

### Feature Selection

We separate the feature selection procedure into two consecutive steps: first, the feature set is reduced using cross-correlation analysis, and then, the remaining features are fed into a sequential feature selection method to find the best subset in terms of classification accuracy.

#### Feature Reduction

We remove highly correlated features, since one of the requirements for the predictor variables to obtain successful linear regression models is to be independent. For this, we use the data of all users together. Table 4 shows the cross-correlation matrix of the phone call features. Highly correlated and significant values tell us that the feature set can and should be reduced to three features (number of phone calls, percent in/out, and, e.g., mean (length)). A similar conclusion can be given for the case of nonlinear HRV features shown in Table 5 where only SD1/SD2 is chosen. The cross-correlation analysis is also applied to other categories of smartphone and HRV features. As a result of the feature reduction, we end up with 13 smartphone and 10 HRV features.

**Table 4:** Correlation matrix of the phone call features

*r*	No. of calls	Sum (calls)	Mean (length)	Std. (length)	% in/out
No. of phone calls	1.00	0.60^+^	0.48^+^	0.65^+^	0.33
Sum (calls)	0.60^+^	1.00	0.91^*^	0.86^*^	0.42^*^
Mean (length)	0.48^+^	0.91^*^	1.00	0.80^*^	0.59^*^
Std. (length)	0.65^+^	0.86^*^	0.80^*^	1.00	0.25^*^
% in/out	0 .33	0.42^*^	0.59^*^	0.25^*^	1.00

$^{+}p < 0.05$; $^{*}p < 0.01$

**Table 5:** Correlation matrix of the nonlinear HRV features

*r*	SD1	SD2	SD1/SD2
SD1	1.00	0.85^*^	0.77^*^
SD2	0.85^*^	1.00	0.81^*^
SD1/SD2	0.77^*^	0.81^*^	1.00

$^{*}p < 0.01$

#### Sequential Feature Selection

For each separate user, we apply the feature selection method, which, starting from an empty set, sequentially selects a subset of features until there is no improvement in prediction. For each candidate feature subset, a 10-fold cross-validation on the user data is performed. For each user, we get a different subset with the corresponding feature importance. Using only smartphone features, the algorithm selects two to five features over all users and four to six features using only HRV features. Table 6 shows the list of selected features ordered by the average importance over all users. The third column shows the result of the feature selection applied to the concatenation of all smartphone and HRV features. From the seven selected features, in the merged case, four features belong to HRV and three belong to smartphone. This ratio gives a qualitative indication that the HRV features are in general more important.

**Table 6:** Selected features from sequential feature selection for smartphone, HRV, and concatenated smartphone + HRV features. They are sorted by the average importance

Order	Smartphone	HRV	All features
1	No. of calls	LF/HF	LF/HF
2	Audio length	SD1/SD2	No. of calls
3	Distance	Sleep length	SD1/SD2
4	Speech energy	RMSSD	Sleep length
5	Mean call length	HRV index	Audio length
6		Mean RR	Distance
7			RMMSD

### Cross-validation

The DS as well as the LTS is directly derived from the output of the logit classification. We therefore use the accuracy of that classification to evaluate the overall system. The self-assessment stress score is mapped into one of the three stress categories. The recognition is correct if the output of the logit classification is the same as the mapped class and wrong otherwise.

**Fig. 8:**
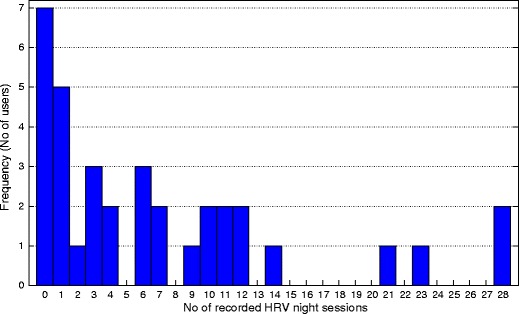
Histogram of the recorded HRV night sessions

Figure 8 shows the histogram of the recorded HRV night sessions. Eleven users have collected 10 and more HRV night sessions, but on the other side, there are 12 users who have only one or less recordings. Since we are interested in combining smartphone data and HRV data, we consider the smartphone data for only those days where HRV recordings are available as well.

#### The General Model vs. User-Specific Models

The general model uses aggregated features of all 35 participants. For a user-specific model, data of one participant which has at least 10 HRV night sessions recorded are used. In both cases, three logit models are trained: the model trained with only smartphone features, $\mathbf {M}_{\mathrm {P}}$; the model trained with only HRV features, $\mathbf {M}_{\mathrm {H}}$; and the model trained with all features, **M**. In the general case, the feature subsets are the columns shown in Table 6. In the user-specific case, the feature set of a specific user results from the previous sequential feature selection for that user.

#### Cross-validation Results

To calculate the classification accuracy of the user-specific models, we employ the leave-one-day-out cross-validation procedure for each user separately. For $\mathbf {M}_{\mathrm {P}}$, we get an average accuracy of 55 %, 59 % for $\mathbf {M}_{\mathrm {H}}$, and 61 % for **M**, for the three-class recognition problem (low, moderate, and high stress) with 40 % (0.7–0.3) baseline. For the general model, we use the leave-one-participant-out cross-validation and get the accuracies of 45 % for $\mathbf {M}_{\mathrm {P}}$, 52 % for $\mathbf {M}_{\mathrm {H}}$, and 53 % for **M**. $\mathbf {M}_{\mathrm {H}}$ outperforms $\mathbf {M}_{\mathrm {P}}$ in both cases by at least 4 %. This finding is aline with the assumption from the feature selection, which says that HRV features are more important. Moreover, the higher decrease of the accuracy for $\mathbf {M}_{\mathrm {P}}$ indicates that the smartphone features are more user sensitive than the HRV features.

### Discussion

#### Study Design

Except for a short description on how to use the app, the participants were not given any other instructions about the study. They used the system in a completely unconstrained environment. When answering to the stress self-assessment question, they were not aware about our definition of work-related stress. For some of them, stress was not necessarily a negative event or feeling. Additionally, what we have not considered so far is the differentiation between the pure working period and the time spent at home in the evening and between workdays and weekend days.

#### Feature Selection

For both smartphone and HRV, at least one feature from each category remains in the feature subsets. But, we can also notice that no feature from accelerometer, address book, calendar, and battery has been selected at all. This is due to the fact that most people do not use their own smartphones for business purposes. The smartphone lies on the desk, and all business contacts and calendar events are stored on the computer. Regarding HRV, the selected features are similar to feature sets found in related works. And, a valuable outcome is that simple features such as the sleep duration and the length of the audio response seem to be important for both modalities.

#### Cross-validation

In the evaluation of the system, we did not consider the ordinal characteristic of the stress classes. The mismatch between classes 0 and 1 or between 1 and 2 should be less penalized than the mismatch between 0 and 2. Compared with the best results achieved in recent works (≈ 80 to 90 %), the accuracy of 61 % seems to be poor at first view. But, considering the fact that we dared to go out of laboratory environment and had no artificial stressors at all during the day, we can conclude that 21 % above chance is reasonable. But, at the same time, we see that there is room for improvement in any aspect, not only including study design and machine learning methods but also incorporating other sensors for stress detection.

## Conclusion and Ongoing Work

We have presented an iPhone-based app, which combines the recording of all available smartphone data with the collection of subjective assessments and voice messages during workday and the recording of HRV data during night. We employed the app to 35 users over 4 months in a real working environment. We analyzed the collected data aiming to find the appropriate feature set for smartphone and HRV in order to build logistic regression models for discriminating stress levels. We have defined two different stress scores, the daily stress score estimating the acute stress level of the previous day and the long-term stress score, which is the accumulated stress over the last days and weeks and which estimates the chronic stress level of a person.

With the cross-correlation analysis, we were able to remove highly correlated features, and the following automatic feature selection led to the best feature subset for each individual user. We noticed that the HRV features are in general more important than the smartphone features.

Finally, we saw that the classification models have to be trained with user-specific data. The model using only HRV data outperforms the model using only smartphone data. The best accuracy, when all features are combined, is 61 % for the three-class recognition problem.

When a user installs the app, the initial period is used to gather objective (smartphone and HRV) and subjective (stress self-assessments) data to train the user-specific logit models. During this calibration phase, the pre-built general models are used to calculate the stress scores. When enough data for that specific user are collected, the user-specific model is built, and from now on, the app switches from training to classification mode. The estimated stress score is daily shown on the smartphone, and the user is asked the self-assessment questions from time to time in order to dynamically update the models and to be able to catch changes in user’s lifestyle.

There are some improvements and extensions that we plan to do next:

### Stress Question

Beside the question in its absolute form, namely “How stressful have you felt today?,” the user is asked additionally two questions in relative form:

- “How stressful have you felt today compared to yesterday (*much less* to *much more*)?”
- “How stressful have you felt today compared to last week (*much less* to *much more*)?”

For the relative questions, the input slider is initially positioned in the middle (0.5). The user can then move the slider towards the *much less* or the *much more* end. By moving the slider towards either of the far edges, the user generates an input that goes up to 75 % ($\alpha $) of the distance remaining from the reference value towards 0 (if slider $<$ 0.5) or towards 1 (if slider $>$ 0.5). More formally, the slider input (in) is turned into an absolute value (out) [0–1] based on the reference value (*ref *) from yesterday or last week and $\alpha = 0.75$ as follows:

$$\text{out} = \begin{cases}\text{ref} - \text{ref}\cdot (1 - \text{in}/0.5) \cdot \alpha &\mbox{if } \text{in} \leq 0.5, \\\text{ref} + (1 - \text{ref}) \cdot ((\text{in} - 0.5)/0.5) \cdot \alpha & \mbox{if } \text{in} >0.5.\end{cases}$$

The overall input of each day is calculated as the average of the three questions, with the above transformation applied to the two relative questions slider inputs beforehand. For the *yesterday* relative question, the reference value ref is the input from yesterday. For the *last week* relative question, the reference value ref is the average of all inputs from the last week. We propose this change as we belief it may help with removing input bias and ensure a more rapid coverage of all three input classes.

### Model Selection

Different other linear and nonlinear classification and regression methods will be evaluated, e.g., SVM or Random Forrest.

### Detect Sleep Stages

We have used the whole HRV night session to extract the time and frequency domain features so far. But, stress is manifested in the RR intervals signal during sleep differently in each sleep stage [6]. Therefore, to have reliable HRV features, the differentiation of the sleep stages would be of benefit. We will evaluate methods to separate the wake stage, the rapid eye movement (REM) stage, and the non-REM stages from each other, based on the distribution of the HF/LF ratio and the typical sleep stage cycles (see, e.g., [27]).

### HRV During Day

We have seen from a related work that measuring HRV during the day is useful as well. Therefore, an HRV session of 15 min before going to sleep and another session of 15 min just after waking up in the morning will be a good supplement to the existing measures during sleep.

### Additional Wearable Device

Beside the Wahoo chest belt, we plan to use other physiological sensors as well. The Empatica (http://www.empatica.com) E2 sensor is a wrist-worn device which is able to read the blood pressure, the skin conductivity, the body temperature, and the body movement. An alternative to that is the Affectiva (http://www.affectiva.com) Q Sensor based on [30].
